# Granuloma Encapsulation Is a Key Factor for Containing Tuberculosis Infection in Minipigs

**DOI:** 10.1371/journal.pone.0010030

**Published:** 2010-04-06

**Authors:** Olga Gil, Ivan Díaz, Cristina Vilaplana, Gustavo Tapia, Jorge Díaz, María Fort, Neus Cáceres, Sergio Pinto, Joan Caylà, Leigh Corner, Mariano Domingo, Pere-Joan Cardona

**Affiliations:** 1 Unitat de Tuberculosi Experimental (UTE), Institut per a la Investigació en Ciències de la Salut Germans Trias i Pujol, Universitat Autònoma de Barcelona, Badalona, Catalonia, Spain; 2 CIBER Enfermedades Respiratorias, Instituto Carlos III, Palma de Mallorca, Spain; 3 Centre de Recerca en Sanitat Animal (CReSA) (UAB-IRTA), Bellaterra, Catalonia, Spain; 4 Pathology Department, Hospital Universitari Germans Trias i Pujol, Badalona, Catalonia, Spain; 5 Tuberculosis Investigation Unit of Barcelona, Servei d'Epidemiologia, Agència de Salut Pública, Barcelona, Catalonia, Spain; 6 School of Agriculture, Food Science and Veterinary Medicine, University College Dublin, Dublin, Ireland; Statens Serum Institute, Denmark

## Abstract

A transthoracic infection involving a low dose of *Mycobacterium tuberculosis* has been used to establish a new model of infection in minipigs. The 20-week monitoring period showed a marked Th1 response and poor humoral response for the whole infection. A detailed histopathological analysis was performed after slicing the formalin-fixed whole lungs of each animal. All lesions were recorded and classified according to their microscopic aspect, their relationship with the intralobular connective network and their degree of maturity in order to obtain a dissemination ratio (DR) between recent and old lesions. CFU counts and evolution of the DR with time showed that the proposed model correlated with a contained infection, decreasing from week 9 onwards. These findings suggest that the infection induces an initial Th1 response, which is followed by local fibrosis and encapsulation of the granulomas, thereby decreasing the onset of new lesions. Two therapeutic strategies were applied in order to understand how they could influence the model. Thus, chemotherapy with isoniazid alone helped to decrease the total number of lesions, despite the increase in DR after week 9, with similar kinetics to those of the control group, whereas addition of a therapeutic *M. tuberculosis* fragment-based vaccine after chemotherapy increased the Th1 and humoral responses, as well as the number of lesions, but decreased the DR. By providing a local pulmonary structure similar to that in humans, the mini-pig model highlights new aspects that could be key to a better understanding tuberculosis infection control in humans.

## Introduction

*Mycobacterium tuberculosis* causes up to 100 million new cases of latent tuberculosis infection (LTBI) every year. The fact that this infection can persist in the host for years explains why LTBI is so prevalent. Indeed, it is estimated that a third of humankind (more than 2.5 billion people) [1] already has LTBI. Progression towards active tuberculosis (TB) (from 5 to 25% of infected people) is relatively low, although even this low percentage represents the induction of 9 million new TB cases every year [2]. Standard treatment for LTBI requires the administration of a potentially hepatotoxic drug, namely isoniazid (INH), for between 6 and 9 months, which results in important compliance problems. Low compliance is the main reason why health systems are reluctant to provide LTBI treatment except in those countries that want to maintain their current low incidence of TB [3] or in the case of HIV co-infection [4]. A shorter non-toxic treatment is therefore crucial to generalize LTBI control and to promote a global decrease in TB incidence, although this necessarily implies a better knowledge of the evolution of LTBI in order to generate new prophylactic and therapeutic approaches.

The diagnosis of LTBI in humans is based on a normal chest X-ray, together with a positive reaction to the tuberculin skin test (TST) and a lack of clinical signs and symptoms suggestive of active tuberculosis [5]. Recently, new tests based on the detection of effector T-cells reacting against antigens secreted by active growing bacilli (ESAT-6/CFP-10) in peripheral blood, the so-called T-cell interferon gamma assay (TIGRAS), have been introduced [6]. Despite this, human LTBI is still mainly diagnosed by indirect methods [5], and little is known about its histopathological evolution.

Different experimental models have been used to better understand LTBI evolution, the most common being those generated in mice. This host is able to control the bacillary load once a strong Th1 immune response has been triggered and a chronic phase generated [7] [8], [9], even with a demonstrated presence of non-replicating bacilli [10], [11]. However, and despite surviving the infection for a long time, progressive infiltration of the lung occurs [12], [13] and all mice finally die from tuberculosis. Murine lesions are characterized by the lack of intragranulomatous necrosis, a discrete fibrotic reaction, lack of encapsulation and a strong lymphocytic presence. The predominance of a strong Th1 response led this response (with very low toxicity for the parenchyma) to be considered an “ideal” response to be reproduced and promoted to avoid evolution towards active TB in humans, as a Th1/Th2 response is seen in humans and human lesions are characterized by a strong tissue toxicity (i.e. necrosis and fibrosis) [14]. The lesions in guinea pig infection (with a relatively low survival time once infected [8]), on the other hand, better resemble human ones as they generate a strong inflammatory response with strong fibrosis (although with no external encapsulation) and strong intragranulomatous necrosis followed by mineralization, mostly in primary lesions, where non-replicating bacilli can be detected [15]. Guinea pigs are also characterized by a strong inflammatory response in the hilar lymph-nodes [16], which appears to be an attempt to avoid the systemic dissemination that would otherwise lead to rapid death, as in the case of humans with detectable bacilli in the spleen [17].

Other animal hosts have been used to better understand human LTBI. Zebra fish infected with *M. marinum*, for example, have provided insights into how mycobacteria exploit the granuloma during the innate immune phase for local expansion and systemic dissemination [18]. Cattle have also been used as a model due to their “human-like” control of the infection and similar immune response in peripheral blood. Indeed, the cattle model is nowadays used as a model to test new prophylactic vaccines against human TB [19]. However, the first evidence for progression from LTBI to active disease, including cavity formation, came from an experimental model using the Cynomolgus macaque [20], which shows a wide spectrum of human-like lesions.

A histopathological description of LTBI in humans was obtained from the studies performed by Canetti in the middle of the last century [21]. This author described the presence of a “benign evolution” of the infection in those necropsy subjects without active TB, with small granulomas encapsulated by a fibrotic ring and mineralized necrosis in the center. This is probably the best histopathological description of LTBI as it shows an efficacious control of the infection that defines this process.

There is still, however, a need to ascertain the role of the parenchyma structure in the evolution of LTBI. The parenchyma in humans and other large animals is subdivided by connective tissue septa into portions of around one to two cubic centimeters, which may play a role in controlling the dissemination of pulmonary lesions [22]. Lungs are lined by a connective tissue band with a mesothelial surface facing the pleural space. As is the case for the lungs of many larger species, such as pigs, cows, and horses, the human lung has extensive interlobular and intralobular connective tissue, which joins the major vessels and the bronchi to the pleural surface. Such structures are not, however, present in smaller species, such as mice, guinea pigs, rabbits or macaques [23].

In light of the factors discussed above, we therefore considered that an experimental model in a large animal was urgently required to gain a better understanding of LTBI. As mentioned above, there is some previous experience using cattle as a model to test new prophylactic vaccines against human TB [19]. We discarded this option, however, as cattle are ruminants and therefore have a different physiology. We therefore decided to develop a swine model, as pigs generate lesions that closely resemble those in human TB patients [24], [25] and there is a precedent regarding the development of an experimental swine model using *M. bovis* [26].

This study presents for the first time the evolution of *M. tuberculosis* infection in minipigs and shows how different treatment regimes can influence it. The results presented herein demonstrate that this model might resembles LTBI in humans as it shows a strong ability to control infection by generating a strong Th1 response together with a strong local response based on the induction of a fibrotic process, which encapsulates the lesions and limits the constant induction of new lesions, and causes intragranulomatous necrosis and calcification of the lesions that entraps and stresses the remaining non-replicating bacilli inside the lesions.

The fact that the local pulmonary structure in this animal model is almost identical to that in humans is of great significance as it may lead to a better understanding of the mechanisms of LTBI induction and allow new prophylactic and therapeutic approaches designed to enhance the strong control of LTBI to be tested.

## Results

### 1. The natural course of the infection in the minipig model: mimicking human LTBI

**Figure 1:**
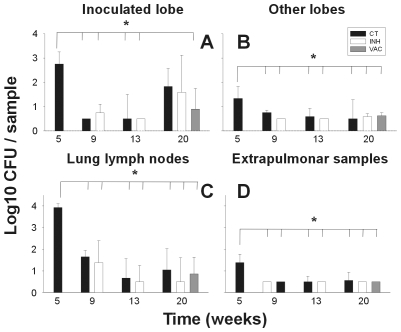
CFU values in the lungs. Data (mean and standard deviation) are presented by comparing an inoculated lobe with others (pictures **A** and **B**), lung lymph nodes (**C**), and extrapulmonary samples (**D**). Every sample collected weighted 2 g. approximately. * signifies a statistically significant difference in the one-way ANOVA test (p<0.005). CT  =  control group; INH  =  chemotherapy with isoniazid; VAC  =  group treated with vaccine therapy.

At the first time point selected to evaluate the minipig model, namely week 5 post-infection, the bacillary load was found to be high at all sites examined: lung, lymph nodes and extrapulmonary samples (spleen, liver, kidneys, extrapulmonary lymph nodes and others). Furthermore, control of the bacillary count tended to stabilize from week 9 to the end of the experiment (Figure 1). Indeed, bacilli could only be detected in the BAL culture for one animal at week 5 and in the blood cultures of another animal at week 9. BAL samples clearly show the presence of foamy macrophages through time.

**Figure 2:**
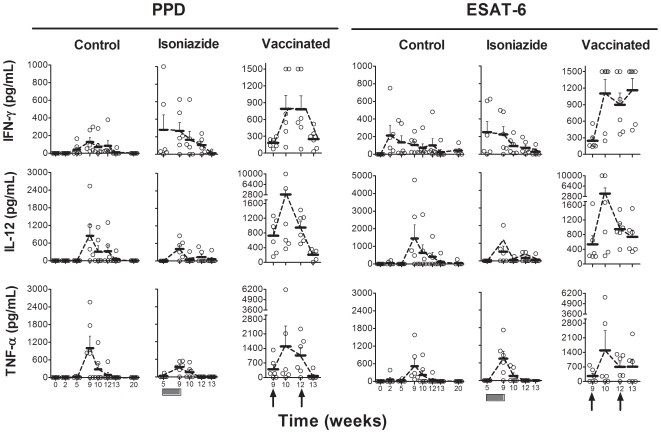
Evaluation of the cellular immune response by ELISA assay. ESAT-6 and PPD-specific IFN-γ, IL-12 and TNF-α production after stimulation of PBMCs, as determined by ELISA assay. Means and standard error of the means (SEM) are drawn in gray and connected by dotted lines. The black boxes show the period of INH treatment and the black arrows the time of therapeutic vaccination.

**Figure 3:**
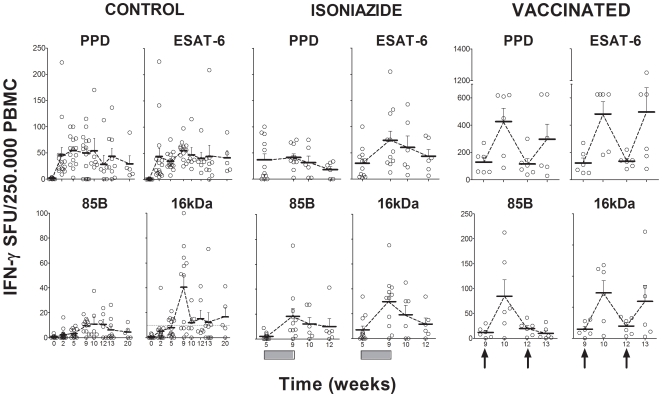
Evaluation of the cellular immune response by ELISPOT assay. Evaluation of ESAT-6, Ag85B, 16 kDa and PPD-specific IFN-γ production after stimulation of PBMCs, as determined by ELISPOT assay. Means and SEM are drawn in black and connected by dotted lines. The black boxes show the period of INH treatment and the black arrows the time of therapeutic vaccination.

**Figure 4:**
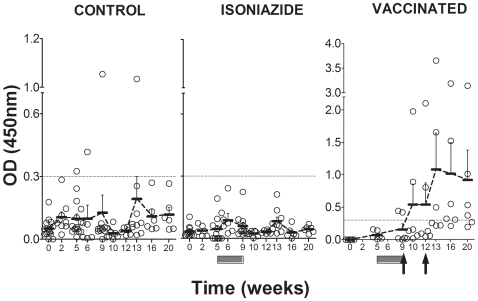
Evaluation of the humoral response. Humoral response against PPD is presented. The dotted line indicates the background threshold. Means and SEM are drawn in black and connected by dotted lines. The dark-grey boxes show the period of INH treatment and the black arrows the time of therapeutic vaccination.

The kinetics of the cellular immune response are shown in Figures 2 and 3. A peak in the evolution was detected 9 weeks after the challenge in non-treated animals, irrespective of the cytokine evaluated or the techniques or antigens used to stimulate the samples. Levels tended to decrease from week 9 onwards to a residual level, which persisted. Statistically significant differences were only encountered in the ELISPOT assay for this time point when the 16 kDa and Ag85B stimulations were used (one-way ANOVA, *p*<0.05). IL-4 and IL-10 levels remained undetectable in all animals. As for the humoral response, only one animal in the control group was found to have serological PPD-specific antibodies from week 13 to the end of the experiment (Figure 4).

Despite the presence of an active infection with a proven continuous residual CFU and cellular immune response, the animals appeared to be in good health, as shown by the welfare checklist conducted as part of the daily clinical monitoring, and continued to gain weight until the end of the experiment. All these data highlight the similarity of our model with the course of human LTBI in terms of the immunological response in peripheral blood, the asymptomatic evolution and the hypothetical control of the CFU concentration in tissue.

### 2. The pathology permitted a classification of granulomas according to their characteristics and showed the role of the intralobular septa network in the encapsulation process

**Figure 5:**
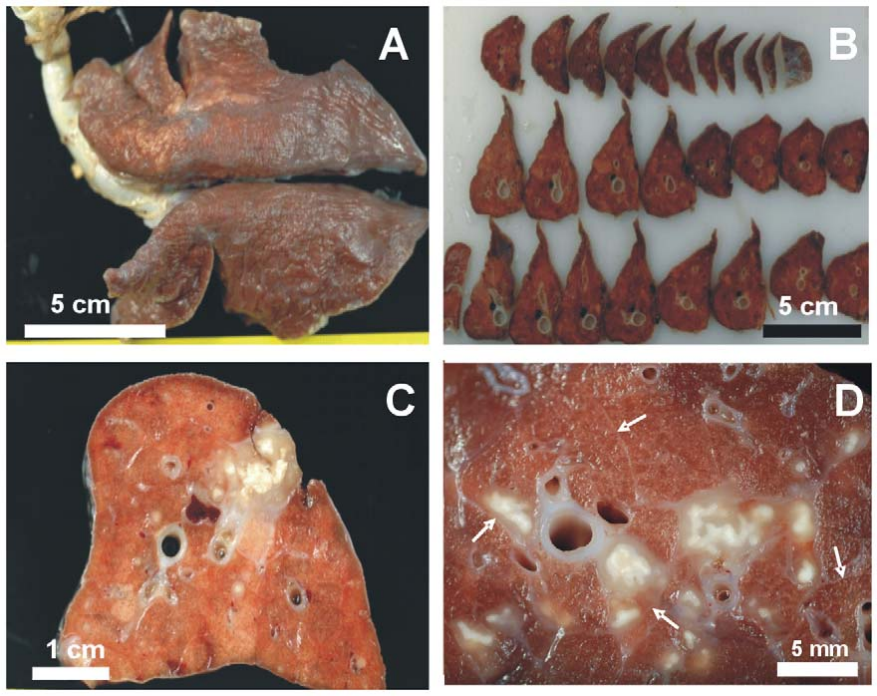
Macroscopic pathology of the whole lung showing the intralobular septa network. Macroscopic pathology of the whole lung (**A**), sliced lobes (**B**) and the inoculation site (**C**). White arrows indicate normal septa and the increased thickness of those surrounding lesions (**D**).

Figure 5 shows the aspect of the lungs once fixed with formalin. Only confluent lesions related to the site of bacillary inoculation (the caudal left lung) could be detected before slicing. Small lesions (0.5–2 mm in diameter) could be seen in the other lobes after careful examination of every slice. Figure 5 also shows the reactivity of the intralobular septa in the vicinity of a lesion. Enlargement of the septa was observed when they were touching a granuloma, even in the presence of a small lesion.

**Figure 6:**
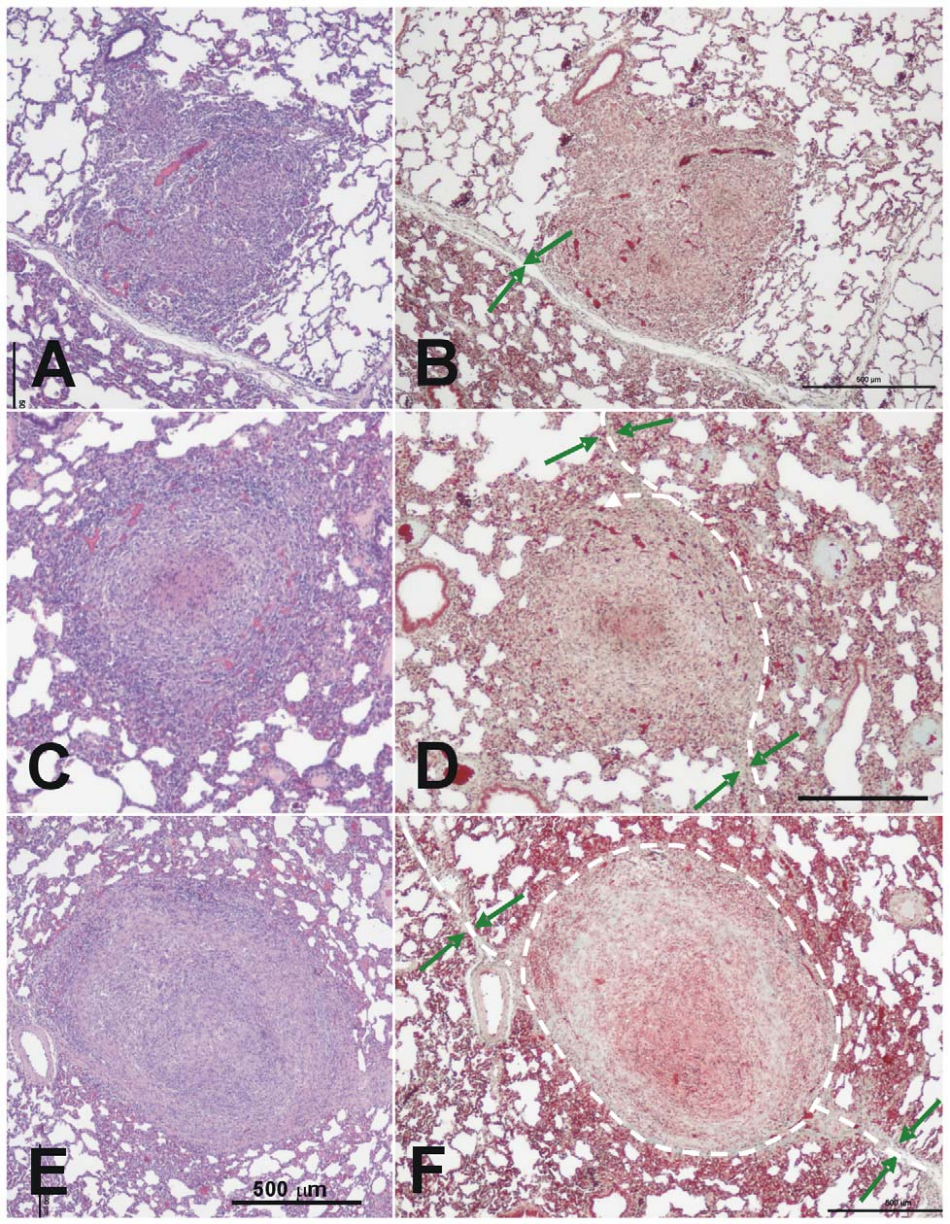
Microscopic evolution of recent lesions, showing the relationship between the granulomas and the intralobular septa. **A–D**: Phase I lesions; **E** and **F**: Phase II lesions. Images **A** and **B** show the initial evolution phase where the granuloma touches an intralobular septa but there is still no fibroblast proliferation. This can be seen in images **C** and **D**, where the septa increase in thickness and start to surround the granuloma, as shown by the white dotted lines. Images **E** and **F** show how the granuloma is finally surrounded by a thick collagenic mantle. Pictures **A**, **C** and **D** were stained with haematoxylin and eosin (H&E), while **B**, **D** and **F** were stained with Masson's trichromic. The original magnification of the large images is ×40 whereas all insets, except **F** (x100), are magnified ×400. The green arrows show the septa and the dotted white lines the trajectory of the capsule.

**Figure 7:**
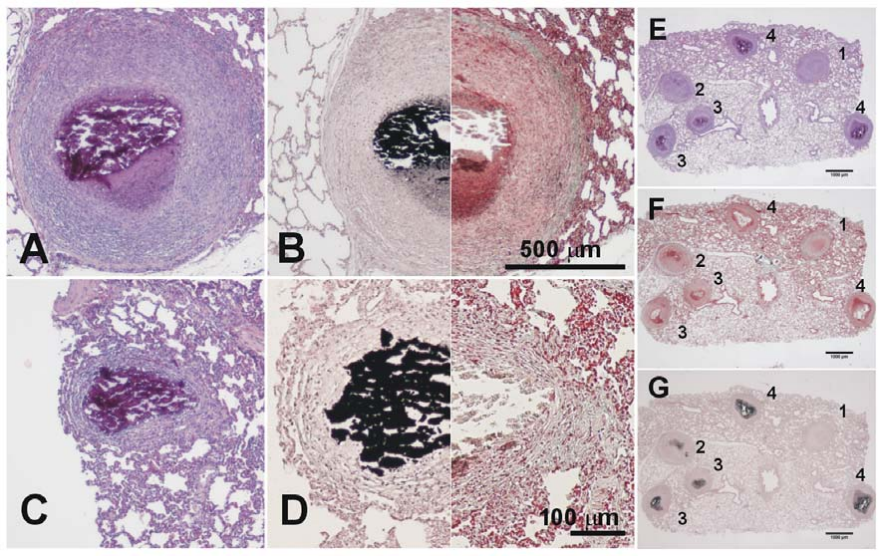
Microscopic evolution of the old lesions. Once the granuloma is structured, the necrotic process starts and calcification appears. Images **A** and **B** (phase III) show a well-structured and encapsulated granuloma with necrotic calcification. Images **C** and **D** show lesions of an advanced evolution (phase IV), with granulomas containing a large amount of calcification and fibrosis. Samples **A** and **C** were stained with H&E, whereas samples **B** and **D** were half stained with Masson's trichromic and von Kossa stain, which shows calcification in black. Pictures **E** to **G** were stained with H&E, Masson's trichromic and von Kossa stain, respectively. Lesions 1 and 2 are phase II lesions and differ only in the initial mineralization seen in lesion 2. The other lesions are all phase III lesions that have progressed differently. Original magnification is ×10.

**Figure 8:**
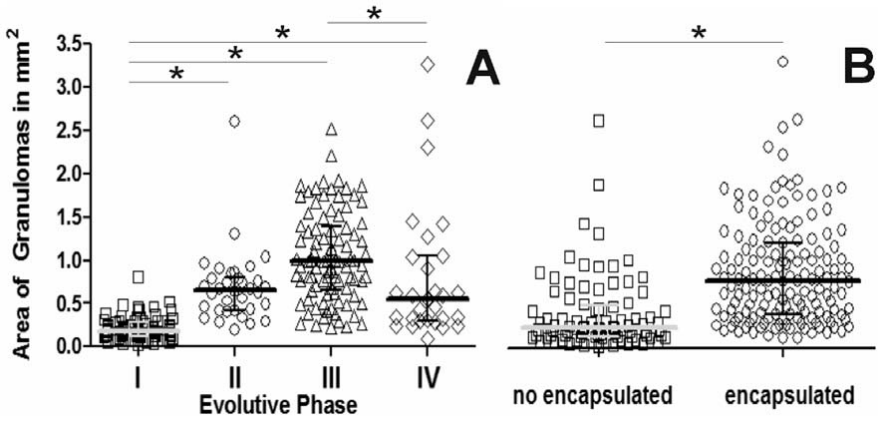
Evolution of the area of granulomas according to their evolutive phase. Individual data are presented in relation to the evolutive state of the lesion (**A**) or its encapsulation status (**B**). Mean and quartiles are presented in each case. Inter-group differences were determined by Dunn's One Anova on ranks test and are marked with * if statistically significant (p<0.05).

A careful examination of 235 lesions permitted us to observe different types of granulomas. Early-phase granulomas seemed barely organized and resembled a mouse-type granuloma, with a non-organized mixture of macrophages, neutrophils and lymphocytes (Figure 6A and B), whereas later-phase lesions (Figures 6C to F) were more organized and offered a more symmetrical sphere-like aspect. A 44.1% of Phase II and a 92.5% of Phase III lesions were encapsulated. These lesions showed a fully organized structure as they mainly contained myofibroblasts along with a very low proportion of lymphocytes and macrophages surrounding a macrophage-based layer containing foamy macrophages and apoptotic cells (mainly neutrophils) around the central necrotic area. In Phase III lesions, the lymphocytes appeared to be mainly accumulated as a thin layer outside the capsule and mineralization was present in all cases, as demonstrated by addition of von Kossa stain (Figure 7). Evolution towards the later phase was mainly based on the increase of the mineralization area and reduction of the surrounding cellularity, together with a reduction in size (Figure 8).

**Figure 9:**
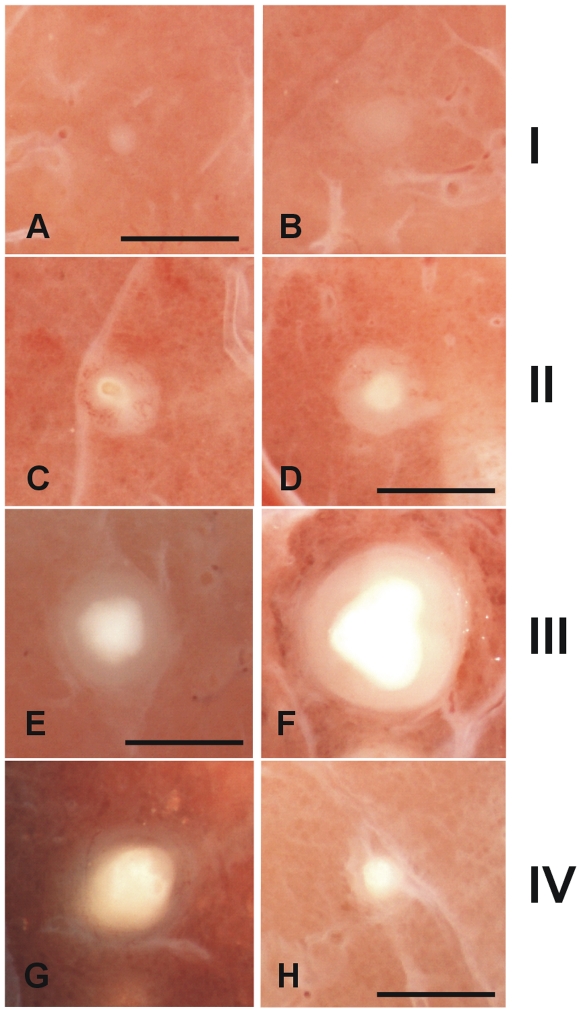
Macroscopic evolution of the lesions. Classification of the fixed pulmonary lesions as they appear under the stereoscopic microscope. Considering the histological evolution of the lesions, and taking into account the sequential appearance of encapsulation and calcification, we have divided the lesions into four phases. Phase I is characterized by the presence of cellular infiltration (**A** and **B**). The intragranulomatous necrosis, which is characterized by the presence of an opaque zone inside the granuloma (**C** and **D**), appears during Phase II and structuration of the lesion starts. Phase III (**E** and **F**) involves the onset of calcification, which gives a shiny aspect to the central opacity, which grows in size. These lesions are characterized by the cartilaginous texture of the lesion when touched with the forceps. Phase IV lesions (**G** and **H**) are characterized by predominance of the calcification and a thin surrounding infiltration. Original magnification is ×10. Scale bar: 1 mm.

According to these data, and after careful comparison of the microscopic preparations and observation with a stereoscopic microscope, we were able to classify the granulomas according to their major recognizable characteristics (Figure 9 and Table 1) as follows: Phase I granulomas were characterized by the presence of an irregular infiltration with no intragranulomatous necrosis. Phase II was considered when structured and the first signs of intragranulomatous necrosis appeared, as revealed by the presence of an opaque area inside the infiltration. Phase III occurred when mineralization dominated the lesion, giving it the appearance of a shiny opaque area, and it became compact and has a cartilaginous consistency when pressed with forceps. We considered this a sign that the lesion had become highly fibrotic, which is not seen in earlier lesions. Finally, phase IV lesions are characterized by the predominance of mineralization with a thin surrounding infiltration. When two or more lesions were found together, they are classified as coalescent.

**Table 1:** Characteristics of the evolution Phase of the lesions.

Phase	Irregular margin	Necrosis	Calcification	Cartilaginous consistency	Halus size in relation to necrosis
**I** *	+	-	-	-	N.E.**
**II**	-	+	+/−	-	+++
**III**	-	++	++	+	++
**IV**	-	++	+++	+	+

* Phase I lesions are subdivided in **a** or **b** regarding its contact with interlobular septae (in the **b** case).

** N.E. means “not evaluable”.

**Figure 10:**
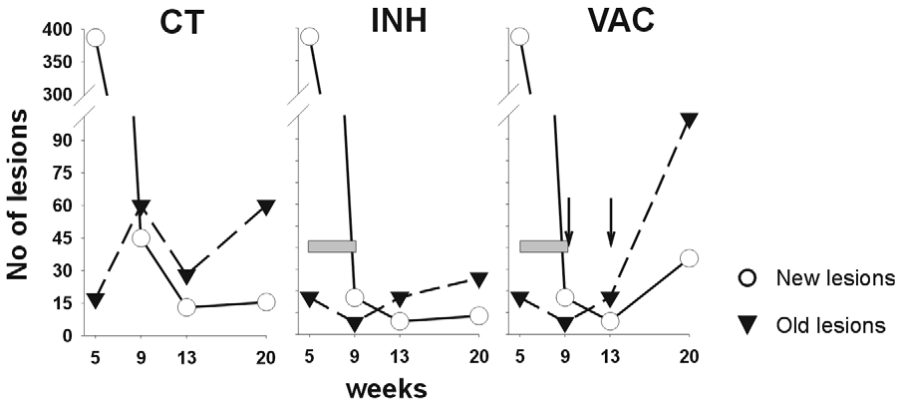
Evolution of recent and old lesions. The results show the median of the values represented in Table 2. The gray bar shows the INH treatment period and vaccine inoculation is represented by the black arrows. CT  =  control group; INH  =  chemotherapy with isoniazid; VAC  =  group treated with vaccine therapy.

After evaluating a total of 4981 lesions (Table 2) we attempted to determine the kinetics of the recent (phase I and II) and older (phase III and IV) lesions (Figure 10). Recent lesions were initially predominant but decreased by week 9, when the number of old ones increased, thus showing their maturation. Both types of lesions tended to decrease from this point onwards, with the older ones predominating. Remarkably, one animal showed no lesions at week 13.

**Table 2:** Characteristics of the lesions.

Week 5							
CONTROL							
	Total lesions			Grouped lesions		DR	
Phase	n	median	25–75 per.	median	25–75 per.	median	25–75 per.
**I**	702	247	*(136.8–328)*	387	*(174–411)*	22.77	*(10.76–44.32)*
**II**	207	64	*(18.25–121)*				
**III**	81	17	*(5.75–50.75)*	17	*(5.75–50.75)*		
**Coalescent**	0	0	*0*				
**IV**	0	0	*0*				
***Total***	***990***	***Median lesions per animal*** and percentiles *(25–75)* = ***404 (180–462)***					

Week 9							
CONTROL							
**I**	62	16	*(10–32.5)*	45	*(18–63.75)*	0.86	*(0.78–0.97)*
**II**	62	29	*(8–31.25)*				
**III**	138	48	*(18.75–72.75)*	60	*(21.75–75.75)*		
**Coalescent**	12	0	*(0–9)*				
**IV**	0	0	*(0–0)*				
***Total***	***274***	***Median lesions per animal*** and percentiles *(25–75)* = ***105 (39.8–140)***					

INH							
**I**	26	13	*(4–22)*	17	*(7–27)*	4.85	*(2.7–7)*
**II**	8	4	*(3–5)*				
**III**	10	5	*(1–9)*	5.5	*(1–10)*		
**Coalescent**	0	0	*(0–0)*				
**IV**	1	0.5	*(0–1)*				
***Total***	***45***	***Median lesions per animal*** and percentiles *(25–75)* = ***22.5 (8–37)***					

Week 13							
CONTROL							
**I**	84	5	*(1.25–60.5)*	13	*(4–153.3)*	0.464	*(0.43–0.87)*
**II**	130	8	*(2.75–92.75)*				
**III**	487	20	*(5.75–354.5)*	28	*(7.75–361.8)*		
**Coalescent**	4	1	*(0.25–2.5)*				
**IV**	11	4	*(1–6.25)*				
***Total***	***716***	***Median lesions per animal*** and percentiles *(25–75)* = ***41 (11.8–515)***					

INH							
**I**	110	2	*(2–81.5)*	6	*(1.5–93.75)*	0.35	*(0.09–1.9)*
**II**	19	4	*(1–12.25)*				
**III**	61	14	*(3.5–38.75)*	17	*(4.25–42.5)*		
**Coalescent**	7	3	*(0.75–3.75)*				
**IV**	0	0	*(0–0)*				
***Total***	***197***	***Median lesions per animal*** and percentiles *(25–75)* = ***23 (5.75–136)***					

Week 20							
CONTROL							
**I**	233	9	*(8–12)*	15.5	*(10–60)*	0.39	*(0.22–0.53)*
**II**	472	6.5	*(3–48)*				
**III**	700	48.5	*(22–178)*	60.5	*(24–270)*		
**Coalescent**	45	3.5	*(1–16)*				
**IV**	109	3	*(1–31)*				
***Total***	***1559***	***Median lesions per animal*** and percentiles *(25–75)* = ***76 (34–330)***					

INH							
**I**	156	5.5	*(1–24)*	8.5	*(4–55)*	0.412	*(0.32–0.83)*
**II**	48	4	*(2–6)*				
**III**	121	11	*(3–26)*	26	*(6–37)*		
**Coalescent**	47	3	*(1–9)*				
**IV**	45	8.5	*(3–11)*				
***Total***	***417***	***Median lesions per animal*** and percentiles *(25–75)* = ***38 (11–155)***					

VACCINATED							
**I**	92	20	*(3–21)*	35	*(7–41)*	0.195	*(0.18–0.32)*
**II**	78	12.5	*(4–18)*				
**III**	389	54	*(15–128)*	99.5	*(36–176)*		
**Coalescent**	26	4.5	*(1–7)*				
**IV**	198	27	*(7–47)*				
**Total**	***783***	***Median lesions per animal*** and percentiles *(25–75)* = ***142.5 (44–214)***					

**Figure 11:**
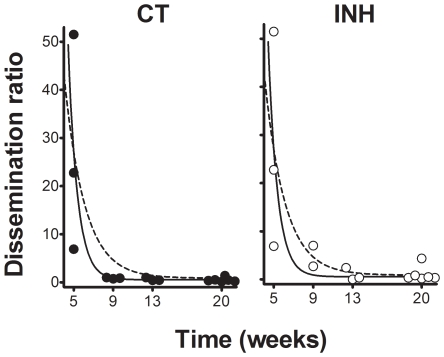
Evolution of the dissemination rate (DR). Individual data points are represented by full or open circles in control (CT) and isoniazid-treated (INH) groups respectively, and are adjusted to an exponential regression. The continuous and dotted lines represents the adjustment for the CT (y = 5620.45^−1.071·x^ + 0.5460) and INH (y = 295.62^−0.4848·x^ +0.8793) groups. Both adjustments were statistically significant (p<0.0001)

In light of the physiological value of the classification between recent and old lesions (recent ones can disseminate bacilli as they are still not well encapsulated), we determined the dissemination ratio (DR) by dividing the number of recent lesions by the older ones in each animal at each time point. Figure 11 shows that evolution of the DR fits a negative exponential regression (p<0.0001), thus demonstrating that, after an initial dissemination period, the generation of new lesions decreased at week 9 to become almost constant (DR of between 0.8 and 0.34) at week 20. This evolution was well supported by the CFU data, which also show a strong control after week 9 (Figure 1).

The presence of acid-fast bacilli inside the granulomas was hardly detected during the whole experiment. The maximum observed was two bacilli per lesion, inside the necrotic tissue in all cases, using the auramine technique (data not shown).

**Figure 12:**
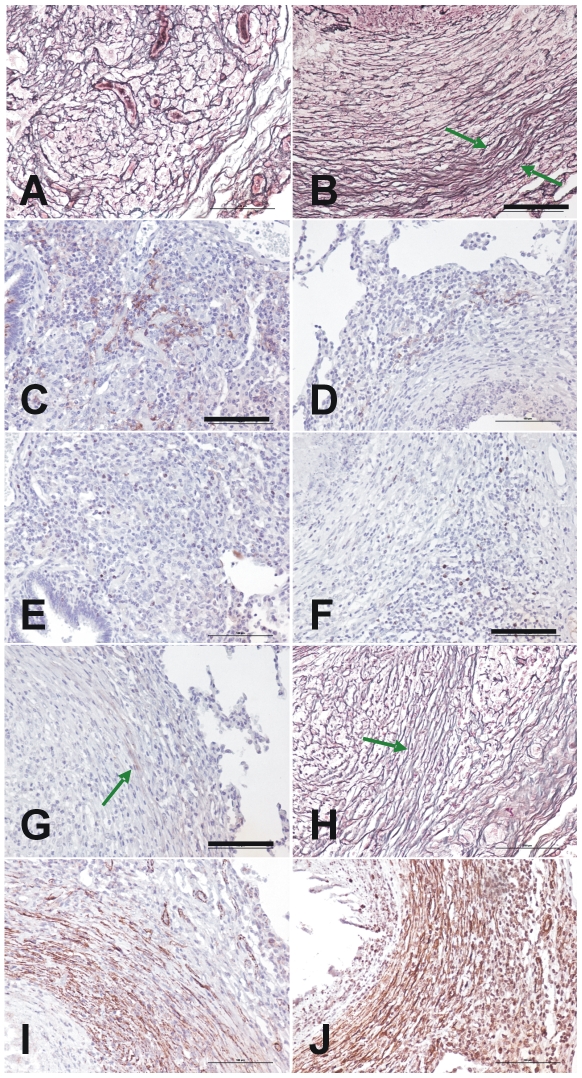
Characterization of the dual fibrotic responses in granuloma evolution. Figures **A**, **B** and **H** show reticulin stain of initial Phase I (**A**) and Phase III granulomas (the peripheral capsule is marked with a green arrow in **B** and **H**). **C** and **D** present immunostaining with anti CD10 and show an increase in the center (**C**) or the periphery (**D**) of Phase I and III granulomas, respectively. **E** and **F** also show the differences between these evolutive phases with the same proliferation pattern stained with Ki 67. **G** shows recognition of the capsule by anti collagen type 1 antibodies, whereas **I** and **J** show the identification of myofibroblasts using anti smooth muscle and anti HHF35 antibodies respectively. Original magnification is ×200. Scale bar: 100 µm.

Two fibrotic processes, the patterns of which are shown in Figure 12, were present during granuloma evolution. The central pattern was initially characterized by the presence of proliferation, active fibroblasts expressing CD10 and a disorganized net of reticulin based on type III collagen. Once phase II appeared, myofibroblasts (recognized by the presence of antibodies against the actin in smooth muscle cells) organized the reticulin network and the cellular proliferation and CD10 expression appeared to be located around the capsule, at the periphery, where both reticulin and Masson's trichromic stains showed thick, wavy collagen fibers. As in the intralobular septa, these were easily recognized by antibodies against collagen type I.

As the encapsulation process was detected in a high number of lesions, an analysis of factors possibly associated with it was performed in order to ascertain its origin; the results are presented in Table 3. At the univariate level, encapsulation was found to be related to the granuloma's evolutive phase, its size, and contact with the intralobular connective network (in particular with the medium-large blood vessels or bronchi, and the intralobular septa; the pleura had no influence). Multivariate analysis confirmed the influence of the evolutive phase and contact between the granuloma and the intralobular septa that join the major vessels and bronchi to the pleura. Although this was observed using a 2D method, the percentage of contact (56.5%) was high enough to predict a much larger percentage if different cuts of the granulomas were assayed using a 3D approach. The area of the granulomas appeared to be less important, although it should be noted that encapsulated lesions appeared to be significantly larger (0.76 vs 0.23 mm^2^, p<0.001) than non-encapsulated ones (Figure 8). Interestingly, a number of Phase I lesions were also found to be encapsulated, a fact that was linked to the presence of neighboring larger granulomas stimulating the same intralobular septa.

**Table 3:** Predictive factors in granuloma encapsulation.

VARIABLE	UNIVARIATE LEVEL			MUTIVARIATE LEVEL	
	Encapsulation	ρ-value	OR (95% CI)	ρ-value	Adjusted OR (95% CI)
	Total (% encapsulated)				
**Evolutive Phase:**					
I	79 (24,1)	<0,001	1	<0,001	1
II	34 (44,1)		2,5 (1,0–6,4)		2,5 (1,0–5,9)
III	93 (92,5)		38,8 (14,2–110,5)		41,6 (16,2–107,3)
IV	29 (93,1)		42,6 (8,6–268,8)		39,2 (8,5–181,7)
**Contact with Pleura:**					
No	217 (62,7)	1	1	-	
Yes	18 (61,1)		0,94 (0,3–2,8)		
**Contact with vessel/bronchi**					
No	79 (45,6)	<0,001	1	-	
Yes	156 (71,2)		3,00 (1,62–5,4)		
**Contact with Septae:**					
No	117 (55,6)	0,03	1	0,045	1
Yes	118 (69,5)		1,8 (1,0–3,2)		2,1 (1.0–4,3)
**Contact with the connective net**					
No	29 (34,5)	0,02	1	-	
Yes	206 (66,5)		3,8 (1,6–9,3)		
**Area size in mm^2^:**					
0–0.23	60 (23,3)	<0,01	1	-	
0.24–0.49	58 (63,8)		5,8 (2,4–14,1)		
0.50–0.97	59 (74,6)		9,6 (3,9–24,5)		
0.98–3.27	58 (89,7)		28,5 (9,2–93,2)		

***OR:*** *Odds ratio.* ***CI:*** *Confidence Interval.*

Previous studies have already differentiated between old (initial or primary) granulomatous lesions and new (secondary) ones, both in rabbits [27] or in guinea-pigs, mainly accordingly to the size of the lesions (larger in the former) and also by the lack of intragranulomatous necrosis in the case of guinea-pigs [28]. However, our study provides, for the first time, a clear histopathological evolution of the lesions in the context of what could be considered as a “human-like” experimental LTBI model. The process described was marked by two kind of fibrotic structures: an internal one based on the production of collagen III organized by myofibroblasts, and an external one directed by the contact with intralobular septa, based on type I collagen. The evolution of new and old lesions provides a dissemination ratio that could be useful for understanding the persistence of LTBI.

### 3. Modification of the natural course of the infection in the minipig model by adding two different therapeutic treatments

Animals were observed daily to ensure their good health according to a pre-established welfare-status schedule. No local effect was detected at the inoculation site at any time (data not shown). Systemic adverse effects were monitored by visual inspection and by monitoring the weight and body temperature. There were no apparent differences among the three experimental groups, although weight tended to increase in all groups and temperature to decrease, whilst remaining in the physiological range. These changes were considered related to the age of the animals.

Neither of the treatments applied resulted in a statistically significant difference in the bacillary counts at any site with respect to the control group.

As shown in Figures 2 and 3, INH treatment did not affect the evolution of the cellular immune response with time, although a slight decrease could be seen from week 13 onwards. In contrast, the ELISPOT assay showed a transient increase in IFN-γ SFU after both the first and second vaccine inoculations (using antigen 85B, PPD, ESAT-6 or 16 kDa as stimuli). This increase was statistically significant when compared to the other two experimental groups. Vaccine therapy also induced a transient increase in IFN-γ, IL-12 and TNF-α one week after the first vaccine inoculation in some animals, as detected using the ELISA technique (Figure 2). This increase was confirmed statistically upon comparison with the other groups. IL-4 and IL-10 levels remained undetectable in all animals.

An increase of serological PPD-specific antibodies was observed after the second vaccine inoculation in all vaccinated animals. Two animals from the vaccinated group already showed positive PPD-specific antibodies in their sera at week 9, before the first inoculation, and these two animals also had the highest amount of antibodies at the end of the study, thus suggesting a boosting phenomenon.

As regards the kinetics of recent and old lesions, INH treatment during weeks 5 to 9 resulted in a reduction of all lesions, thereby reflecting a decrease in the maturation process. Remarkably, only two lesions could be detected in one animal belonging to the INH group at week 13 (Figure 12). Interestingly, vaccine therapy increased both types of lesions, with the number of old lesions showing a higher increase. This treatment also decreased the CFU numbers of the inoculated lobe (Figure 1).

Although we have already shown that DR decreased sharply from week 9 to a residual constant value until the end of the experiment for the non-treated animals, INH treatment stopped this decrease, apparently by stopping the progression of lesions to phases III and IV but without changing the overall evolution defined by the control group. Interestingly, vaccine therapy modified this evolution by reducing the DR to 0.195 at week 20, a fact strongly related to the increased number of old lesions (Figure 10).

In conclusion, treatment of the infection with short-term chemotherapy reduced the number of lesions but appears to also reduce their maturation towards old lesions. In contrast, the addition of a therapeutic vaccination increased the number of lesions, mainly the old ones, tends to reduce the CFU counts, and could be linked to an increase in the (cellular and humoral) immunological responses induced.

## Discussion

Little is known about the histopathological evolution of human LTBI, although some findings have helped to determine the evolution of the lesions [21], [29].

In general, a strong inflammatory response is induced, with the presence of giant multinuclear cells, a strong fibrotic response encapsulating the lesion, and a large intragranulomatous necrosis, which finally calcifies over time. The presence of bacilli in these lesions is seldom observed. As a chest X-ray is able to detect old calcified lesions, it is traditionally considered that, upon infection with *M. tuberculosis*, an initial dissemination of lesions, which might evolve towards calcification, takes place before the immune response is triggered [17]. It was demonstrated a long time ago that bacilli can persist in a latent stage in a small percentage (around 1%) of these evolved lesions [30]. This is the origin of the traditional theory, which postulates that these bacilli can resuscitate after some type of local immunodepression and produce active TB [29]. This theory has, however, been superseded to some extent by other less disseminated and/or less accepted theories developed to explain LTBI: external reinfection as the most probable cause of TB in adults [21]; *M. tuberculosis* infection undergoing a constant process of autoinoculation, with bacilli constantly being drained out of the lesions and inducing febrile episodes [31], [32]; and the more recent hypothesis that considers LTBI to be a consequence of a constant endogenous reinfection process [33].

As the “perfect” animal model that mimics LTBI has still not been found, we present a new animal model using minipigs. In this study we describe for the first time a comprehensive evolution of the lesions caused by *M. tuberculosis* in a large animal that might fit the traditional view of control of this infection in humans, which leads to an LTBI. This is seen in a context characterized by a maintained Th1 response, control of the bacillary load, the constant induction of recent lesions, and their resolution (towards old lesions) as a result of a strong local fibrotic response in which the local intralobular septa seem to play a crucial role in granuloma encapsulation. This response seems to play a major role in the evolution of granulomas by curtailing the dissemination of bacilli towards the alveolar space, thereby explaining the sudden drop in the induction of new lesions 9 weeks after the challenge. As this dissemination still exists, albeit at a low level, these findings also support the Dynamic Hypothesis, which postulates that maintenance of an LTBI requires a constant endogenous reinfection [33], and might explain the success of humans in controlling TB infections and the low progression towards active TB.

Interestingly, the initial granulomatous dissemination observed also fits with the work developed in the zebrafish model infected with *M. marinum*, where infected macrophages from primary granulomas could disseminate to initiate new secondary granulomas during the innate immune phase of the infection [18].

The initial lesions in this model are similar to the initial murine lesions, in other words a mixture of neutrophils, macrophages and lymphocytes without much organization, with the periphery open to the alveolar spaces. However, very few acid-fast bacilli are seen in minipigs, whereas they are highly abundant in mice, thus reflecting a similar degree of “human-like” low tolerance to the bacilli as such a low concentration is enough to trigger a strong granulomatous reaction. As in mice, a close relation to the alveolar spaces still exists in this phase, thus allowing the constant drainage of infected foamy macrophages towards this space [13], [34]. As in guinea pigs or larger mammals, this drainage is very effective as the alveolar spaces are large enough not to retain them locally, as is the case in mice. This phase is, however, rather limited in time, as also demonstrated by the scarcity of foamy macrophages detected in the BAL. This phenomenon could be a consequence of the entrapment of foamy macrophages in the granuloma during encapsulation and might therefore play a role in the generation of intragranulomatous necrosis [35], [36].

Our data permit the lesions to be classified and show that the evolution of the granulomas is marked by fibroblast proliferation and transformation into myofibroblasts, which contract the lesions by structuring the reticulin fibers, thereby allowing the original structure to be maintained against external mechanical forces. The granuloma itself develops with a spherical form due to an energy-minimization process [37], [38]. This follows the usual healing process in the lung once a lesion has been induced. Upon onset of infection, intracellular bacillary growth mainly causes necrosis of the infected macrophages, thus maintaining the inflammatory response. In this scenario, secretion of TNF by macrophages is crucial for both fibrin and collagen deposition, which results in a provisional extracellular matrix [39]. This inflammatory response also generates apoptotic neutrophils and macrophages which, once phagocytosed, stimulate the production of TGF-β and thus stop the inflammatory response and provoke an anti-inflammatory process, thereby promoting fibrosis [40], [41], [42]. TGF-β also promotes fibroblast accumulation and, together with mechanical stress, induces the transformation into myofibroblasts [37] [43], whose presence has already been linked to mycobacterial granulomas [44].

The animal model studied herein also shows two sclerotic processes that have classically been described in the human “benign” evolution of the infection [21], namely an increase in fibroblast proliferation, which leads to the internal accumulation of myofibroblasts, and the formation of a capsule around the granuloma, as a continuum of the interlobular septum, once the granuloma has reached it. The similarities with human granulomas might then be a consequence of the presence of intralobular septa in minipigs. These structures are highly sensitive to mechanical stress and react to it with fibroblast proliferation and thickening after collagen production [22]. This capsule appears to be vital to prevent the constant drainage of latent bacilli towards the alveolar spaces, as animals that are not able to structure it, such as mice and guinea pigs, constantly have infected foamy macrophages in the BAL [13], [34], [45]. This process of healing and avoiding constant bacillary dissemination has been known for some time and was the rationale behind the most successful pre-chemotherapy TB therapy, namely collapse therapy, which effectively reduced the appearance of new lesions, avoided liquefaction and also increased local fibrosis and healing by increasing the stasis of the blood circulation [21], [32].

It has been speculated that both fibrosis and the anti-inflammatory response restrict the arrival of new macrophages that are able to phagocytose the apoptotic bodies [46], thus allowing them to accumulate and favoring the mineralization process. Calcification could also be promoted by the destruction of foamy macrophages, which could be both a source of intragranulomatous necrosis [35], [36] and of phosphatidylserine, a molecule that is able to promote the local accumulation of calcium and phosphate ions along with an increase in the local pH [47], [48] [49], [50]. Both these factors could induce a multi-stress scenario against the bacilli, thereby contributing to control of the bacillary load, as has already been demonstrated in the guinea-pig model [15].

As regards the influence of different treatment regimes on the course of the infection, the effect of INH treatment on the evolution of the lesions is worthy of comment. Thus, although this treatment prevents the induction of new lesions and reduces their concentration, it also diminishes the concentration of old lesions, thus indicating a reduction in the fibrosis process that is probably related to a reduction in the inflammatory process, as discussed previously [51]. This could mean that some type of inflammatory process is needed to induce the fibrotic process, possibly by causing enough apoptotic bodies to enhance TGF-β production, although this would need to be demonstrated experimentally. Interestingly, the addition of a therapeutic vaccine has the opposite effect: it increases the number of lesions, particularly old ones. This could reflect a proinflammatory process that might involve the quicker detection and control of new lesions, due to the increased cellular and humoral response, and which would finally increase the fibrotic effect. Previous studies with this particular vaccine have already revealed its ability to elicit a combined Th1-Th2-Th3 response [52], a cellular immunity characterized by an increase in the level of Th1 cells that are able to recognize replicating and non-replicating bacilli [45] and a protective humoral response [53]. This increase in the number of lesions might result from a protective role, as supported by the reduction in both the number of CFUs in the inoculated lobe and the dissemination rate.

Overall, the data presented herein suggest a scenario very similar to human LTBI, and could support the idea of a constant reinfection process maintaining the LTBI [33]. It is well known that the chance of developing active TB after infection decreases exponentially with time [54]. Concurrently, our data also show how the induction of new lesions decreases suddenly after an initial dissemination process and remains at a constant residual level, thus reducing the chance of progression towards active TB [17]. Finally, these results are also in agreement with the constant presence of ESAT-6-specific IFN-γ spot-forming units in the peripheral blood of LTBI human subjects, thereby revealing the presence of short-lived specific effector T-cells [6]. As ESAT-6 is produced by growing bacilli [55], the explanation for the constant presence of these cells might be related to the constant development of new lesions, as demonstrated in the experimental model proposed here. These new lesions would be so small (around 0.5–1 mm) that they would not be observable in the chest X-ray, therefore their presence would be absolutely compatible with a diagnosis of LTBI [33].

The “secret” to controlling *M. tuberculosis* infection might therefore lie in both avoiding the drainage of non-replicating bacilli (by encapsulating the lesion) and stopping the inflammatory response. This would prevent any phagocytosis of the remaining bacilli, thus stopping their growth. This is a crucial point as extracellular multiplication of *M. tuberculosis in vivo* has only been seen in liquefacted lesions [56], which are not present in this scenario. In addition, this anti-inflammatory effect could favour mineralization of the necrotic tissue, which might be deleterious for the healing process but would further stress any remaining non-replicating bacilli.

The high number of parallelisms found between this infection model and the process thought to occur in humans makes it especially useful for further studies devoted to understanding the evolution of LTBI. Achieving a course of the infection that might be similar to the human one, including the monitoring of the immune responses, also provides an opportunity to not only help new preventive and therapeutic approaches against it to be designed, but to be tested with some reliability, thereby offering a completely new field for future studies.

## Materials and Methods

### 1. Experimental Design

#### Animals

A total of 36 1.5-month-old female specific pathogen-free (*spf*) Göttingen minipig® were obtained from Ellegaard (Dalmose, Denmark) and fed with maintenance diet 127 (SAFE, Augy, France). All animals were provided with a highly detailed certificate and were routinely checked for infection with more than 40 infectious agents, including 24 bacteria, 12 viruses, 4 parasites and 3 fungi.

#### Infection and experimental groups

The animals were anesthetized by intramuscular injection of 10 mg/kg of ketamine, 2 mg/kg of xylacine and 2 mg/kg of azaperone before infection with 2×10^3^ CFUs of *M. tuberculosis* diluted in 2 mL of saline. The *Mycobacterium tuberculosis* strain used was the H37Rv Pasteur strain, grown in Proskauer Beck medium containing 0.01% Tween 80 to mid-log phase and stored at −70°C in 2 mL aliquots until being used to infect the animals. The transthoracic method was preferred to an aerosol as it is safer for the professionals responsible for infecting the animals. The transthoracic infection was performed with an 18-gauge Tuohy epidural needle (length: 90 mm) (Viggon S.A, Paterna, Spain) between the 8th and 9th rib of the left lung. Three experimental groups were established according to the treatment received: infected non-treated (control; n = 15); treated with INH alone (INH; n = 12); and treated with INH plus two doses of *M. tuberculosis* fragment-based vaccine (vaccinated; n = 6). INH (300 mg; Cemidon, Alcala Farma, Madrid, Spain) was administered intramuscularly into the cervical region twice a week for four weeks (weeks 5–9), with three days between injections. This schedule was chosen to determine the effects of INH during its most efficacious period, on the basis of previous bactericidal activity studies [57], assuming that the CFU control would start at week 5 after challenge, as previously demonstrated in guinea pigs [58]. Moreover, this schedule had already been found to be effective in large animals [59]. The therapeutic vaccine used was RUTI®, a vaccine manufactured by Archivel Farma, s.l. (Badalona, Catalonia, Spain), under GMP standards. RUTI® vaccine is based on *M. tuberculosis* fragments, detoxified and liposomed as published elsewhere [52]. The vaccine is still in clinical development and not commercially available, thus was kindly provided by the manufacturer. RUTI® was administered twice, at weeks 9 and 12, 21 days apart, according to previous protocols [59].

#### Ethics

All experimental procedures were approved and supervised by the Animal Care Committee of the Universitat Autònoma de Barcelona in accordance with current EU legislation regarding the protection of experimental animals. All experiments were performed in a BL3 facility inside the CReSA building at the Universitat Autònoma de Barcelona. Animals were observed daily according to a welfare schedule and check list (approved by the CRESA Animal Welfare Committee) to monitor the clinical aspects after the infection and to ensure the safety of the treatments tested. They were also weighed every week in order to monitor their health status.

### 2. Post-mortem analysis

#### Post-mortem examination

The animals were euthanised with an intravenous overdose of pentobarbital sodium at week 20 post-innoculation (Vetoquinol, Madrid, Spain). The minipigs were subjected to a detailed post-mortem examination [60]. All obvious lymph nodes present at each site were collected, including the axilar (right and left), inguinal (right and left), mesenteric, hepatic, middle retropharyngeal (right and left), tonsil (right and left), tracheobronchial and caudal mediastinal lymph nodes. A small portion of each lymph node was removed for histology and the remainder retained for bacteriology. Bacteriology tissue samples were collected using an aseptic technique. Gross lesions from the lungs were detected by observation or palpation, recorded and collected for histopathology and culture. If no lung lesions were identified, a sample (approx. 2 g) was collected from the dorsal margin of each lobe for culture. The remainder of the lungs was fixed in formalin in all cases for subsequent slicing at 2-mm intervals. The cut surfaces were further examined for gross lesions and collected for histopathology. Each slice was also carefully examined for microscopic lesions under a binocular stereoscopic microscope SMZ800 (Nikon Instruments Inc., Tokyo, Japan).

#### Bronchoalveolar lavage (BAL)

After sacrifice and aseptic removal of the lungs, a total of 200 mL of physiological serum was introduced through the trachea and recovered by aspiration with a 100 mL syringe. The volume recovered was then divided into two tubes: one to determine the CFU and the other for cytology. The latter tube was centrifuged at 300 g for 15 minutes and the sample stained with Oil Red and examined by electron microscopy to identify foamy macrophages.

#### Bacterial load

Samples of lung lobes and pulmonary and extrapulmonary lymph nodes from each animal were extracted to determine the bacterial burden. Lung tissue and BAL samples were homogenized and decontaminated according to a previously reported procedure [61], whereas lymph node samples were not. The number of viable bacteria was determined by plating serial dilutions of whole organ homogenates and BAL on nutrient Middlebrook 7H11 agar (BD Diagnostics, Spark, USA) and Lowenstein-Jensen medium (Biomedics, Madrid, Spain). Bacterial colonies were counted after incubation for 28 days at 37°C. Blood cultures were performed until week 13 by inoculating 5 mL of whole blood to BacT/ALERT MB flasks (Biomerieux, Marcy L'Etolile, France). These samples were incubated for 30 days at 37°C before being considered negative, as recommended by the manufacturer.

#### Histopathology and histometry

Histopathology samples were fixed in 10% buffered formalin, sectioned at 4 µm and stained with haematoxylin-eosin and Masson trichromic, and for acid-fast bacteria following the Ziehl–Neelsen and auramine methods. Von Kossa stain was also used to evaluate the presence of calcified material in the necrotic tissue [62]. A reticulin silver stain was run to determine the organisation of collagen in the granuloma. Reticulin mainly stains the thin fibrils of type III collagen, although type I collagen can also be stained when organized into thick fibrils assembled in parallel bundles [62]. The small and large axis of each granuloma were recorded from a random sample of Masson trichromic cuts using a DS-Fi1 camera attached to an Eclipse 50i microscope using the NIS-Elements D version 3.0x software package (Nikon Instruments Inc., Tokyo, Japan). The area of 235 granulomas was obtained using the formula of the area of an oval; Pi*a*b, where a = half length of major axis (horizontal) and b = half length of minor axis (vertical).

#### Immunohistochemistry

For immunohistochemical analysis, 4-µm consecutive sections were deparaffinized in xylene and rehydrated with graded alcohol. Heat-induced antigen retrieval was carried out in an autoclave with citrate buffer at pH 6.0. Endogenous peroxidase was blocked with 5% hydrogen peroxide and detection was performed using an UltraVIEW DAB Detection universal Kit in a Benchmark XT device (Roche Diagnostics, Germany). Commercially available anti-human antibodies against CD10 (clone 56C6, 1/20, Novocastra-UK), Ki67 (Clone MIB-1, 1/800, Dako-Denmark), Collagen I (ab6308, 1/500, Abcam-UK), Actin (Clone HHF35, 1/300, Dako-USA), and Alpha Smooth Muscle Actin (Clone αsm, 1/200, Novocastra-UK) were obtained from the indicated suppliers.

#### Semi-thin slices from lung granuloma and BAL

Lung granulomas were placed in Eppendorf tubes containing the fixation solution 1x (2.5% glutaraldehyde and 2% paraformaldehyde in phosphate buffer 0.1 M) and kept at 4–8°C for at least 24 h. BAL tubes were centrifuged at 300 g for 10 minutes, then resuspended in 0.5 mL of PBS and 0.5 mL of the fixation solution 2x and kept at 4–8°C for between 2 and 24 h. The BAL samples were again centrifuged at 300 g for 10 minutes, then the supernatant was removed carefully, the fixation solution 1x added slowly and the samples kept at 4–8°C. Granuloma and BAL samples were then post-fixed in osmium tetroxide, dehydrated and embedded in epoxy resin. Semi-thin sections were stained with toluidine blue as described elsewhere [34].

### 3. Evaluation of the immune response

The evolution of the cellular immunological response was monitored by stimulation of PBMCs using different specific stimuli, as well as detection of several cytokines in plasma. The humoral response was also evaluated.

#### Immunological responses after stimulation of PBMCs

Blood was collected using heparinized blood tubes at the time points described above. Peripheral blood mononuclear cells (PBMCs) were separated from whole blood by density-gradient centrifugation with a Histopaque 1077 (Sigma) and washed. Trypan blue was added to assess their viability.

The abundance of antigen-specific IFN-γ secreting cells in the PBMCs was analyzed by an ELISPOT assay using commercial mABs (Porcine IFN-γ P2G10 and biotin P2C11, BD Biosciences Pharmingen) according to a previously reported method [63]. Briefly, Costar 3590 ELISA plates (Corning, New York, USA) were coated overnight with 5 µg/ml P2G10 capture antibody diluted in carbonate/bicarbonate buffer. The plates were then washed and blocked for 1 h at 37°C with 150 µl PBS containing 1% BSA. After removal of the blocking solution, 5×10^5^ viable PBMCs were dispensed per well and stimulated with PPD, ESAT-6, Ag 85B, Ag 16 kDa (all at a final concentration of 10 µg/ml) or BCG (5×10^5^ CFUs/ml).

PPD RT-49 and BCG were obtained from the Statens Serum Institute (SSI, Copenhagen, Denmark). The recombinant *M. tuberculosis* antigens ESAT-6 (Rv3875), Ag 85B (Rv1886c) and Ag 16 kDa (Rv2031c) were purchased from Lionex Diagnostics and Therapeutics GmbH (Braunschweig, Germany).

Unstimulated cells and phytohaemagglutinin (PHA)-stimulated controls (10 µg/ml) were also included in both experiments. After 20 h incubation at 37°C in a humidified atmosphere of 5% CO_2_, the cells were removed and the biotinylated P2C11 detection antibody was added at 0.5 µg/ml and incubated for 1 h at 37°C. The reaction was revealed by sequential incubation of plates with streptavidin-peroxidase and insoluble 3,3′5,5′-tetramethylbenzidine (TMB; Calbiochem, Nottingham, UK). To calculate the antigen-specific frequencies of IFN-γ secreting cells, also known as spot-forming units (SFUs), the number of spots in unstimulated wells was subtracted from those in antigen-stimulated wells. The abundance of antigen-specific IFN-γ secreting cells was expressed as number of responding cells from a total of 10^6^ PBMC.

To analyze cell-culture supernatants after stimulation of mini-pig PBMCs with different specific antigens, cells were plated (5×10^6^ per well) in 48-well plates and mock-stimulated or stimulated with PPD, ESAT-6 (10 µl/ml) or PHA. After incubation for 20 h at 37°C in a humidified atmosphere of 5% CO_2_, cell culture supernatants were collected and frozen at −80°C until needed. Capture ELISAs for IFN-γ, TNF-α, IL-4, IL-10 and IL-12 were performed as mentioned above.

#### Serum cytokines

Cytokine concentration in sera was determined at weeks 0, 2, 5, 9, 10, 12, 13 and 20 using commercial pairs of antibodies (Porcine IFN-γ, BD Biosciences Pharmingen; TNF-α, IL-4, IL-10 and IL-12; R&D Systems) according to previously reported procedures [63] and the manufacturer's instructions. The cut-off point for each ELISA was calculated as the mean+3SD OD of negative controls. The production of each cytokine was calculated using the linear-regression formula based on the ODs of the cytokine standards provided by the manufacturer and expressed as pg/ml.

#### Humoral response

Costar 3590 ELISA plates (Corning, New York, USA) were coated overnight with 4 µg/ml of PPD RT-49 diluted in carbonate/bicarbonate buffer. They were then washed five times with PBS plus 1% Tween 20 and blocked for 45 minutes at 37°C with 150 µl PBS plus 0.5% casein. After washing, 1/200 diluted sera in PBS plus 0.5% casein was added and incubated for 1 h at 37°C. The plates were further washed and the conjugate (diluted in PBS with 1% Tween 20) added. The conjugate was prepared with 1/10000 protein A-HRP (Sigma, Madrid, Spain) and 1/5000 protein G-HRP (Bio-Rad Laboratories, El Prat del Llobregat, Spain). The reaction was revealed with soluble TMB (Calbiochem, Nottingham, UK) and stopped 20 minutes later with 0.5 M H_2_SO_4_. The plates were read at 450 nm using a conventional spectrophotometer. The unspecific response between serum and conjugate was determined for each sample using a control to which PPD RT-49 had not been added. Results were obtained by analyzing the sera in duplicate and subtracting the unspecific response. The cut-off point was calculated as the mean+3SD OD of unspecific responses.

### 4. Statistical analysis

A descriptive study of the qualitative and quantitative variables collected was carried out in order to characterise the study population. Frequency distributions and medians for quantitative variables were calculated. Proportions were compared between groups using the chi-squared and, when pertinent, the two-sided Fisher test. Measures of association were calculated using *odds ratios* (OR) along with their 95% confidence intervals (CI). The factors associated with encapsulation were analysed using logistic regression (stepwise method) including the variables associated with a p-value of less than 0.15 at the univariate level in the model. A p-value of less than 0.05 was considered significant. The test of Hosmer and Lemeshow was used to check the goodness-of-fit of the models. Analyses were conducted using the SPSS statistical package, version 13.0 (SPSS Inc, Chicago, IL, USA).
